# Exposomic Determinants in Heart Failure Among Older Adults – Mechanistic Links, Clinical Evidence, and Future Perspectives

**DOI:** 10.1007/s11897-026-00781-w

**Published:** 2026-10-24

**Authors:** Omar Hahad, Thomas Münzel, Jiayin Zheng, Arijan Valar, Yanislav Hrytseniuk, Boopathy Ramasamy, Amit Ghosh, Monalisa Mishra, Trupti Das, Philipp Lurz, Andreas Daiber, Marin Kuntic

**Affiliations:** 1 Department of Cardiology, University Medical Center of the Johannes Gutenberg-University Mainz, Mainz, Germany; 2 German Center for Cardiovascular Research (DZHK), Partner Site Rhine- Main, Mainz, Germany; 3 CSIR-Institute of Minerals and Materials Technology, Bhubaneswar, India; 4 All India Institute of Medical Sciences, Bhubaneswar, India; 5 National Institute of Technology, Rourkela, India; 6 Center for Thrombosis and Hemostasis (CTH), University Medical Center of the Johannes Gutenberg-University Mainz, Mainz, Germany

**Keywords:** Heart failure, Ageing, Exposome, Cardiovascular disease

## Abstract

**Purpose of Review:**

Heart failure (HF) in older adults emerges from the interplay between biological aging and the totality of environmental and behavioural exposures conceptualized as the exposome. Traditional cardiovascular risk factors and intrinsic cardiac pathology only partially explain HF burden, as chronic exposures to air pollution, traffic noise, temperature extremes, toxic metals and chemicals, artificial light at night, and adverse social environments, along with low greenness and walkability, substantially influence HF incidence, decompensations, and prognosis in older patients with multiple comorbidities.

**Recent Findings:**

Recently, epidemiological studies have associated many of the exposomic factors with HF incidence and morbidity. Age‑related impairments of the myocardium and vasculature make them particularly vulnerable to external stressors. Mechanistically, aging, HF, and environmental factors converge on shared pathways including, oxidative stress and mitochondrial dysfunction, inflammaging, impaired autophagy and proteostasis, genomic and epigenetic alterations, cellular senescence, dysregulated neurohormonal and circadian signalling, and fibrosis with maladaptive remodelling. Through these mechanisms exposome chronically accelerates cardiovascular aging and lowers the threshold for HF onset and progression.

**Summary:**

This review considers a shift from an organ‑centred view of HF in older adults to a systems‑level, life‑course perspective that explicitly incorporates exposomic determinants, and argues that reducing harmful exposures while promoting green space, physical activity, walkability, and healthy dietary patterns should be integral to HF prevention and management strategies in aging societies.

## Introduction

Heart failure (HF) is a complex chronic clinical syndrome in which the heart cannot pump enough blood to meet the body’s need for oxygen supply, leading to symptoms such as shortness of breath, fatigue, and fluid retention in the lungs and peripheral tissues [1, 2]. This impaired pumping can result from a weakened or stiff ventricle (reduced or preserved ejection fraction) and is commonly driven by conditions like coronary artery disease, hypertension, cardiomyopathies, and valvular disease [3].

The exposome concept provides a framework to explore the totality of all non‑genetic factors, e.g. exposures and lifestyle, an individual experiences from conception onward, including chemical, physical, biological, behavioural, and social factors [4]. It complements the genome by capturing how air pollution, traffic noise, climate, diet, lifestyle, workplace, and neighbourhood conditions interact over time to influence disease risk and health trajectories across the life course of an individual.

Environmental factors can influence both the development and progression of HF by increasing cardiovascular stress and accelerating underlying pathology [5]. Long-term exposure to air pollution and traffic noise raises the risk of incident HF and hospitalizations, while heat waves, cold spells, indoor pollutants, and adverse neighbourhood or social conditions can trigger decompensations in people with established chronic disease [6–8]. These exposures act through pathways such as oxidative stress, systemic inflammation, endothelial dysfunction, blood pressure and autonomic changes, and disruption of sleep and circadian rhythms, which are particularly relevant in older, comorbid patients [8–10].

## Why are Older HF Patients Vulnerable

The pathophysiology of aging is usually depicted by the seven pillars of ageing [11], which include: impaired stress adaptation, epigenetic modifications, increased inflammation, increased macromolecular damage, impaired metabolism, declining proteostasis, and impaired stem cell function and regenerative capacity. All these biological processes, which are modulated in aging, are susceptible to environmental and behavioural stressors which comprise the exposome [12]. Environmental noise causes impairment of the stress response through overstimulation of the sympathetic nervous system (SNS) and the hypothalamic-pituitary-adrenal (HPA) axes, leading to increased inflammation [13, 14]. Air pollution exposure, specifically particulate matter [15], artificial light at night (ALAN), lack of green spaces, and poor social environment can also impair the stress response through the same pathways [16–20]. Epigenetic modifications, macromolecular damage, impaired metabolism and proteostasis have previously been observed in the pathophysiology of metabolic syndrome, which is associated with air pollution, chemical pollution (soil and water) [21], and poor diet [22–24]. Interestingly, environmental noise was also associated with metabolic changes [25], demonstrating that different environmental determinants can converge to influence similar biological process. Underlying biochemical mechanisms by which the biological process of ageing influences the myocardium and vasculature, such as increased oxidative stress, impaired nitric oxide (·NO) signalling, endothelial dysfunction, microvascular rarefaction, and reduced autonomic flexibility, are all amplified by environmental stressors [12]. Together, these biological processes and underlying biochemical mechanisms all contribute to the pathophysiology of HF [10, 26, 27], providing a strong link between aging, exposomic determinants and HF. This interconnectedness of pathophysiological mechanisms between HF, aging and environmental stressors makes older HF patients especially susceptible to exposomic determinants.

**Fig. 1:**
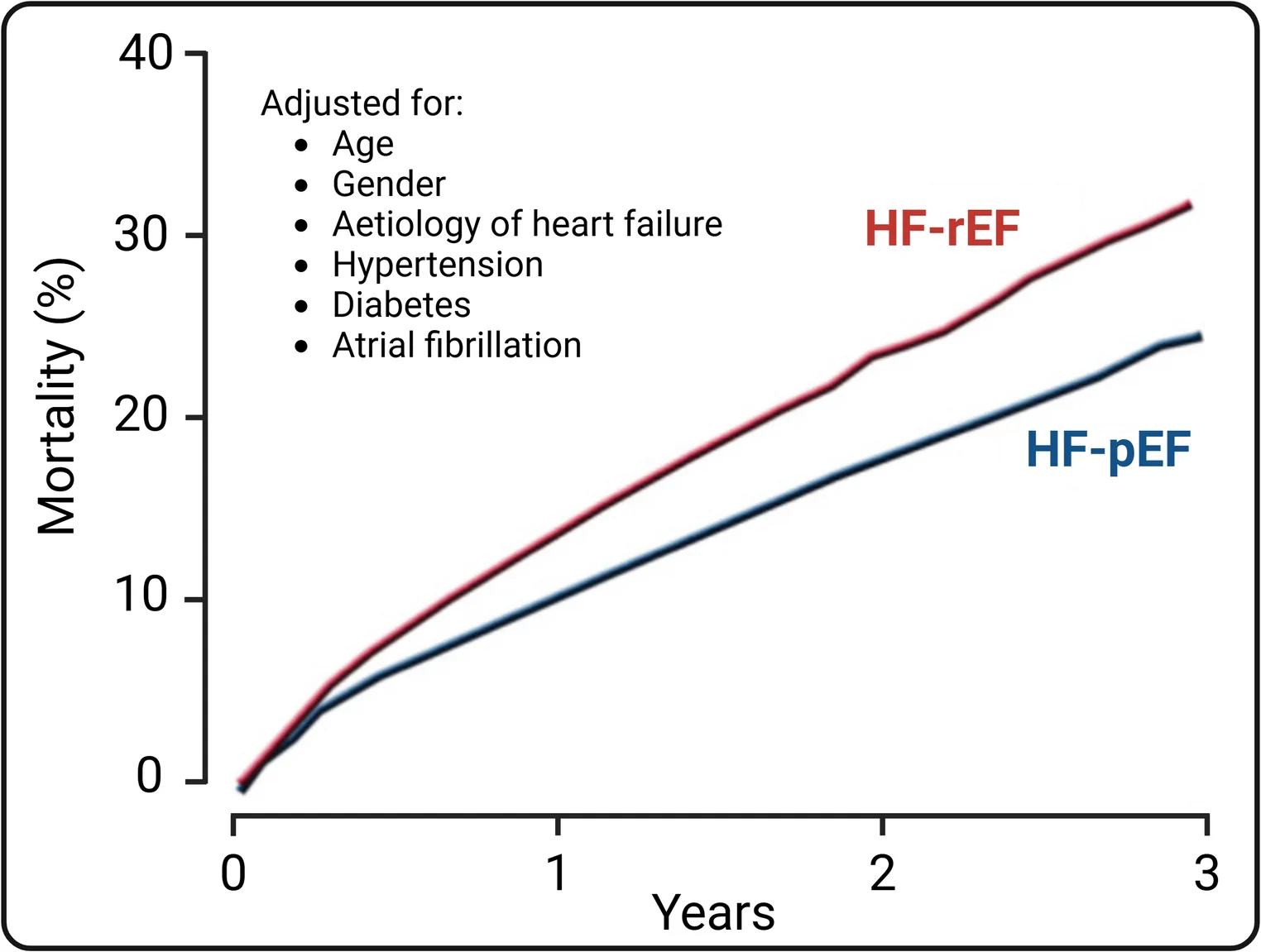
Mortality for patients with heart failure with preserved ejection fraction (HF-pEF) and heart failure with reduced ejection fraction (HF-rEF) adjusted for age, gender, aetiology of heart failure, hypertension, diabetes, and atrial fibrillation (reused with permission from [27] and [31])

Another very important reason why older HF patients are more susceptible to environmental stressors is the presence of comorbidities [28, 29]. Older HF patients have high prevalence of comorbidities like hypertension, chronic kidney disease, chronic obstructive pulmonary disease (COPD), diabetes and polypharmacy [12]. Exposures such as PM₂.₅ and heat can precipitate decompensation through blood pressure changes, arrhythmias, fluid shifts, and increased susceptibility to infection [5]. Also, aging impairs the neurovascular interface in the heart, reducing central regulation and lowering the ability of the heart to adapt to different stimuli [30]. Overall, HF is a major cardiovascular driver of mortality with no cure but only delaying therapies, which makes it a central cause of global burden of disease and for preventive measures also by targeting environmental and lifestyle risk factors (Fig. 1).

## Heart Failure and Exposomic Determinants

**Fig. 2:**
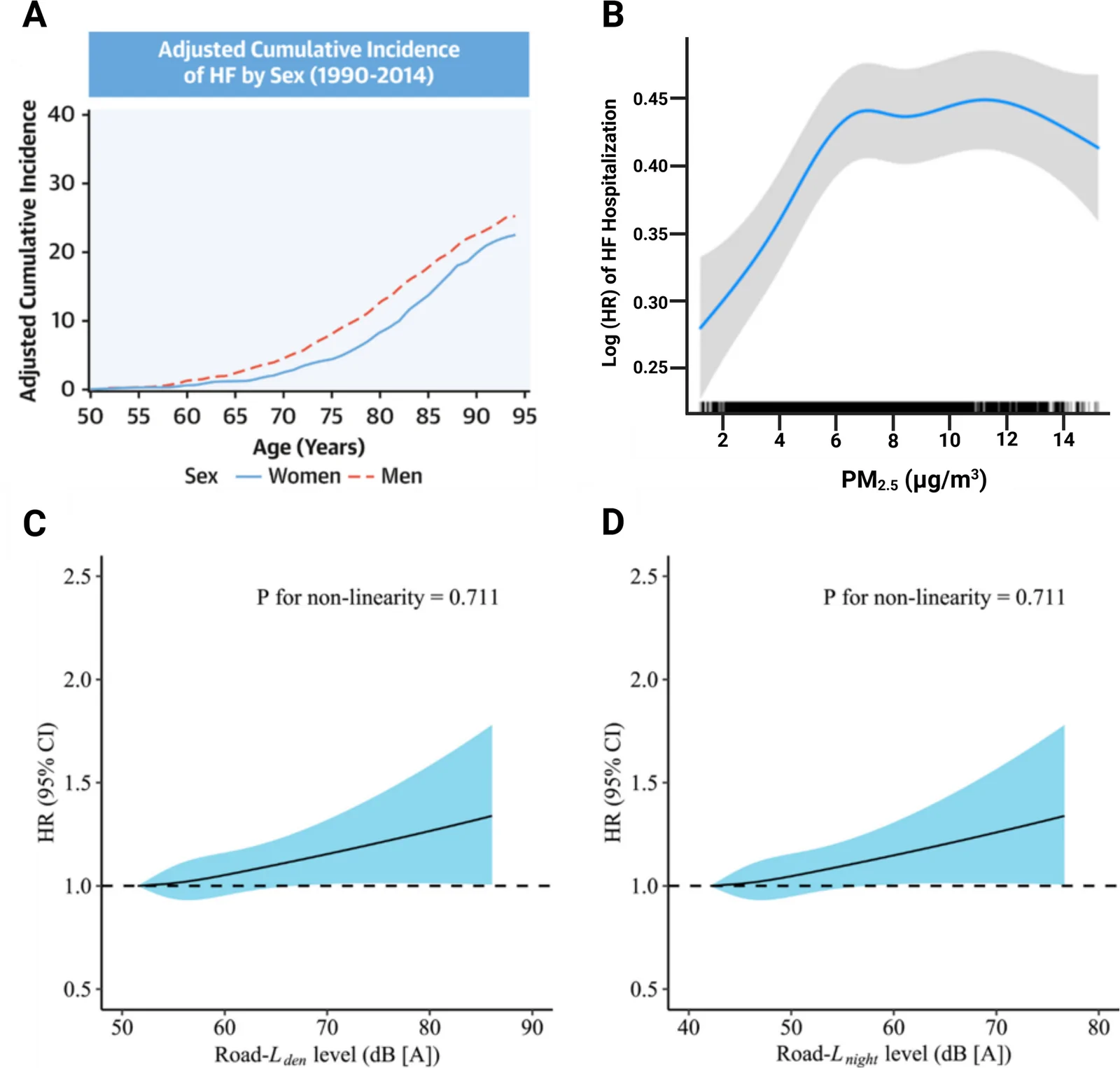
Age and selected exposomic determinants across the life course and risk of heart failure**.** (**A**) Adjusted cumulative incidence of HF by sex (reused from [130] with permission). (**B**) Association between PM_2.5_ and HF hospital admissions (reused from [38] under CC BY-NC-ND 4.0 licence). Hazard rations for incident HF in association with road traffic noise during the day (**C**) and during the night (**D**) (reused from [46] with permission)

**Fig. 3:**
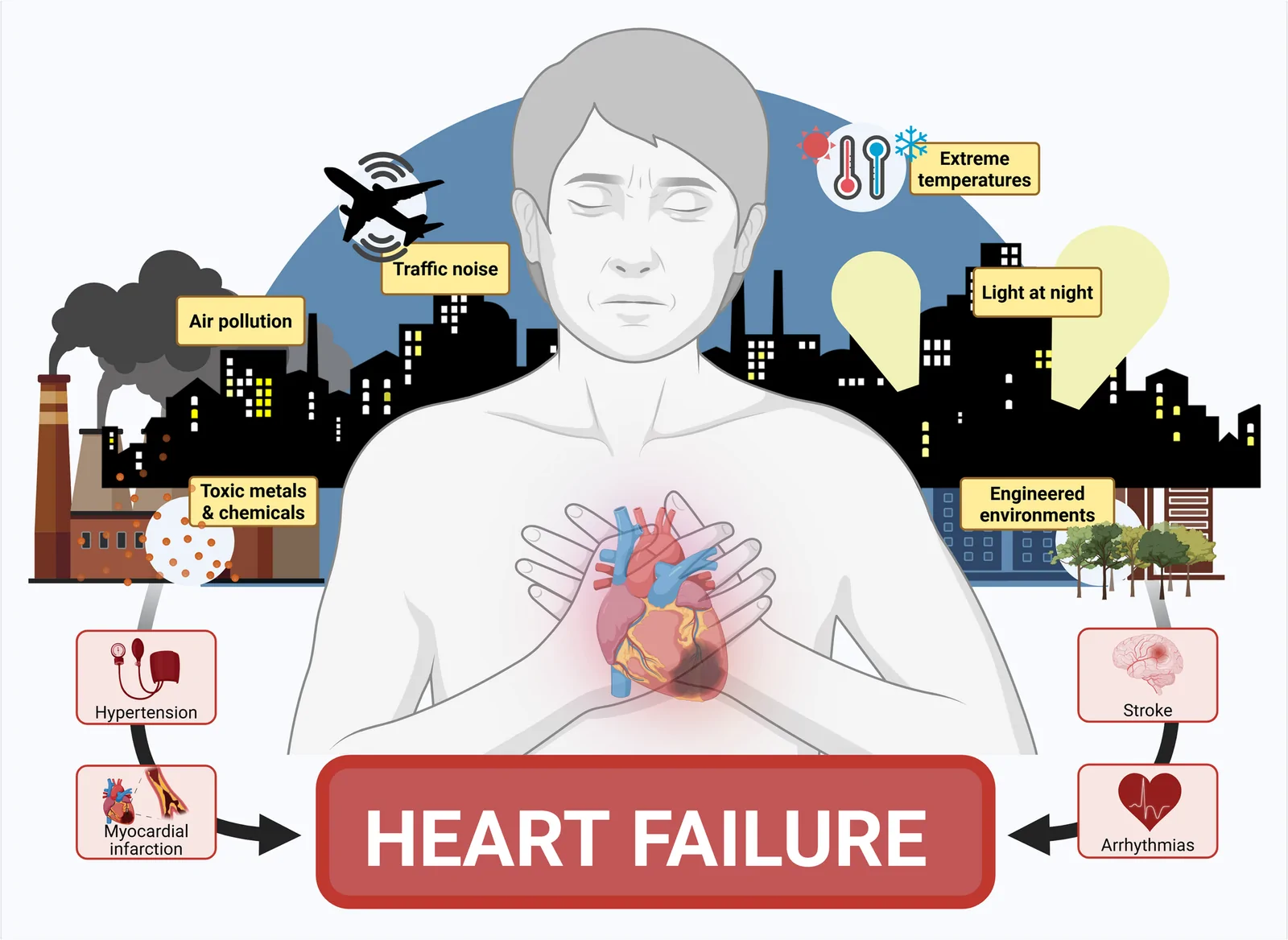
Association between exposomic determinants and heart failure. Clinical studies from large cohorts have identified associations between exposomic determinants and heart failure, especially in old age. Created with BioRender.com

Association between HF and age, together with selected exposomic determinants is shown in Fig. 2. An overview of the effects of exposomic determinants on HF is shown in Fig. 3.

### Air Pollution

Air pollution was previously associated with HF in large cohorts, and interventional studies. Short-term increases in major air pollutants (PM_2.5_, PM_10_, CO, SO_2_, NO_2_) cause small but consistent rises in HF hospitalizations and deaths [32], while long-term exposure substantially increases HF incidence [33–37]. Large cohort studies (UK Biobank, Ontario, US HF cohorts, Medicare beneficiaries, heart transplant recipients) show that chronic exposure to PM_2.5_, PM_10_, NO_2,_ and O_3_ is associated with higher HF risk, hospital utilisation, readmissions, and mortality [35, 38, 39]. For instance, in a nationwide Danish cohort of > 22,000 female nurses (aged > 44 years), a 5.1 µg/m^3^ increase in 3-year mean PM^2.5^ exposure was associated with a 17% higher risk of incident HF (HR 1.17, 95% CI 1.01–1.36), whereas an 8.6 µg/m^3^ increase in NO_2_ was associated with a 10% higher HF risk (HR 1.10, 95% CI 0.99–1.22) [6]. In the FILTER-HF randomized trial, controlled diesel exhaust exposure acutely worsened endothelial function, raised BNP, and reduced exercise capacity in HF patients, and these effects were significantly mitigated by respiratory filtration, highlighting filtration as a potential protective strategy [40]. Additionally, some studies have found a stronger correlation between selected air pollutants and HF in older patients [41, 42], although many cardiovascular health outcomes have been more strongly associated with air pollution constituents in elderly populations [5]. Indoor exposures are especially relevant in geriatrics because older adults spend more time indoors and may be exposed to combustion products, VOCs, and poor ventilation, which have been linked to higher cardiovascular risk and HF events [5].

Studies in animal models also indicate that air pollution can precipitate or aggravate HF–like phenotypes, particularly when cardiac vulnerability is already present. In hypertensive or HF models, particulate matter exposure worsens ventricular dysfunction, promotes hypertrophy and fibrosis, and increases decompensation and arrhythmia risk, reducing cardiac reserve in ways that mirror clinical HF [43, 44].

### Traffic Noise

Air pollution and traffic noise are often confounded in urban environments. Many of the cohort studies investigating the effect of air pollution have additionally assessed the association between traffic noise and HF incidence. A large systematic review and meta-analysis of prospective cohort studies (*n* ≈ 8.6 million participants) demonstrated that higher road traffic noise exposure is significantly associated with an increased incidence of HF, with a hazard ratio (HR) of 1.16 in the older population (> 55 years) and an estimated 7% increase in risk per 10 dB increment above 50 dB [45]. A nationwide study using the UK Biobank found that for every 10 dB(A) increase in road traffic noise, the risk of incident HF increased by an average of 11% among participants with a mean age of 56.5 years [46]. Likewise, a case-control study from Germany reported that the risk for HF and hypertensive heart disease increased with road traffic noise among individuals older than 60 years [47]. The same study also identified a significant increase in the odds of HF alone among individuals with a mean age of 57.5 years, associated with rising sound pressure levels from aircraft, road, and railway noise individually. In contrast, data from the Ontario cohort study indicated that there is a strong association between road traffic noise and congestive HF incidence, although the risk is attenuated with increased age [48]. In a nationwide Danish cohort of more than 2.4 million adults aged ≥ 50 years, long-term exposure to transportation noise was associated with an increased risk of incident HF [49]. For road traffic noise, each 10 dB increase was associated with a higher risk of HF both at the most exposed façade (HR 1.039, 95% CI 1.033–1.045) and at the least exposed façade (HR 1.087, 95% CI 1.073–1.101). Railway noise was also associated with HF when assessed at the least exposed façade, with an HR of 1.028 (95% CI 1.004–1.053), whereas no association was observed at the most exposed façade (HR 0.999, 95% CI 0.988–1.011). The retrospective analysis of 487 HF patients (mean age: M = 65 years; F = 66 years) showed that individuals residing in high-noise areas (> 55 dB) exhibited increased HF risk factors like elevated systolic and diastolic blood pressure, increased heart and respiratory rates and higher circulating levels of B-type natriuretic peptide (BNP) compared to those living in lower-noise environments (< 55 dB) [50].

### Heat, Cold, and Climate

In the context of ongoing climate change and the increasing frequency and intensity of extreme weather events, there is growing evidence that exposure to extreme heat or cold increases the risk of adverse cardiovascular outcomes, with HF exhibiting the highest proportional excess mortality among all cardiovascular subtypes during both temperature extremes [51–53]. Healthy young adults effectively dissipate excess body heat through compensatory responses such as skin vasodilation and increased heart rate when exposed to heat. However, these responses are diminished in older adults, who experience greater increases in core temperature and greater cardiovascular strain under the same conditions [54, 55]. In HF patients, who are predominantly elderly, this age-related impairment in thermoregulation is further compounded by reduced cardiac reserve [56], heat-induced sympathetic activation, and an increased susceptibility to arrhythmias [57], making them the most vulnerable group during extreme heat [58]. Although extreme heat events produce steeper short-term increases in cardiovascular mortality risk [51], cold exposure accounts for a far greater cumulative burden: the Multi-Country Multi-City (MCC) Collaborative Research Network analysis demonstrated that cold-related cardiovascular deaths far exceed heat-related ones [59]. In addition, a nationwide Swedish time-stratified case-crossover study including 250,640 patients with HF (mean age at death 84.3 ± 9.4 years), demonstrated that short-term exposure to both low and high ambient temperatures was associated with increased mortality. A U-shaped association between temperature and mortality was observed. Low temperatures were associated with increased all-cause mortality (OR 1.130, 95% CI 1.074–1.189) and cardiovascular mortality (OR 1.160, 95% CI 1.083–1.242). High temperatures were also associated with increased all-cause mortality (OR 1.054, 95% CI 1.017–1.093), while cardiovascular mortality increased during the later study period (2014–2021; OR 1.084, 95% CI 1.014–1.159) [60]. Mechanistically, cold triggers sympathetic activation and peripheral vasoconstriction, increasing afterload beyond the failing heart’s capacity to compensate, resulting in well-documented winter peaks in HF hospitalizations and mortality [61, 62], with a Canadian population-based study demonstrating that each 1 °C decline in mean temperature over the preceding seven days was associated with a 0.7% increase in the risk of HF events [63].

Additionally, extreme temperature vulnerability is compounded by the therapeutic paradox that routine medications such as diuretics and beta-blockers may themselves impair thermoregulation [57], worsening the climate change-induced cardiovascular disease.

### Toxic Metals and Chemicals

There is consensus that exposure to non-essential metals is associated with increased incidence of CVD [64, 65]. Several large cohort epidemiological studies confirmed that increased blood and urinary levels of toxic metals are associated with increased HF incidence [65–67]. Meta-analysis from the Multi-Ethnic Study of Atherosclerosis [32], Strong Heart Study (SHS), and Hortega cohorts demonstrated that higher urinary cadmium, zinc, molybdenum, and copper individually and as a mixture are significantly associated with increased HF incidence [68]. National Health and Nutrition Examination Survey (NHANES) data showed a significant association of higher blood cadmium and urinary cadmium, tungsten, cobalt, and iodine with increased HF incidence [69, 70]. In addition, the mean age of the participants in the NHANES study influenced the association between urinary arsenic levels and HF, with stronger association found in older cohort participants [70, 71]. In the Malmö Diet and Cancer (MDC) cohort, participants in the highest quartile of blood cadmium had an almost twofold higher risk of incident HF compared with those in the lowest quartile (HR 1.95, 95% CI 1.02–3.71), with the association being confined to participants aged 57–67 years (HR 2.20, 95% CI 1.07–4.50), whereas no association was observed in those aged 46–57 years [66]. Similarly, in the Danish Diet, Cancer and Health (DCH) cohort restricted to never-smokers, higher urinary cadmium concentrations were associated with increased HF incidence (HR 1.10, 95% CI 1.00–1.20 per interquartile-range increase), with a markedly stronger association among men (HR 1.50, 95% CI 1.20–1.90 per interquartile-range increase; highest vs. lowest quartile: aHR 2.00, 95% CI 1.20–3.20) [67]. Interestingly, both MDC and Danish Diet, Cancer and Health (DCH) cohort showed a significant positive association between cadmium level (blood and urinary, respectively) and HF incidence only among male participants [66, 67]. In contrast, a study focused on elderly women with a mean age of 75.2 years in Australia, also found a significant association between higher urinary cadmium and increased incidence of HF and death from HF [72]. In addition, studies in non-elderly people showed that increased levels of blood lead and urinary cadmium, arsenic, and uranium are associated with LV hypertrophy and/or impaired left ventricular function, which might promote HF in the future [73–75]. Specifically, exposure to lead and cadmium was associated with impaired left ventricular systolic function, including reductions in global and regional longitudinal strain, suggesting that even low-level exposure may contribute to subclinical myocardial dysfunction preceding HF [75]. Similarly, higher urinary arsenic concentrations were associated with increased left ventricular wall thickness, left ventricular hypertrophy, and adverse alterations in cardiac geometry in young adults, with stronger associations observed among individuals with elevated blood pressure [73].

There is a substantial body of evidence that environmental, occupational, and dietary pesticide exposure is associated with increased incidence of CVD and CVD mortality [76, 77]. Study based on the SIMPLER cohort (women aged 49–83 years and males aged 45–79 years, follow-up up to 13 years) found a significant association of higher dietary intake of polychlorinated biphenyls (PCBs) with increased HF risk [78]. Retrospective cohort study in Taiwan revealed an association of organophosphate pesticide poisoning with a higher incidence rate of CHF, CAD, and arrhythmia, with most of the effect attributable to participants of ≤ 49 years [79]. Besides, several cohort studies identified an association of increased pesticide exposure with other cardiovascular events, which serve as risk factors of HF. An association of hexachlorobenzene and p,p’-DDE (metabolite of DDT) serum levels with increased left ventricular wall thickness [80] and hypertension [81] were observed in a large cohort. Many other cohort studies confirmed the associations between pesticide exposure and cardiovascular outcomes [82–87]. Although it is clear that pesticide use is associated with HF, the specific impact in aged populations is still understudied.

### Light Pollution

ALAN exerts its negative effects mostly through modulation of the circadian rhythms. Disruption of circadian rhythms is closely linked to metabolic dysfunction and increased CVD risk, with changes in metabolic pathways acting as a main signal for perturbation of internal homeostasis [88–91]. ALAN suppresses melatonin, promotes oxidative stress, inflammation, and autonomic imbalance, and induces chromatin remodelling and insulin resistance, producing metabolic and vascular phenotypes that resemble those caused by other environmental stressors (such as air pollution), although caused via partly distinct molecular pathways [92, 93]. ALAN also fragments sleep and shortens sleep duration, and sleep impairment is a known contributor to hypertension, obesity, and insulin resistance [94, 95]. Epidemiological studies from large cohorts such as PURSE-HIS and the UK Biobank show associations between night-time light and higher blood pressure, impaired vascular function, and increased incidence of HF, coronary disease, stroke, and other cardiometabolic outcomes [96, 97]. For instance, in a prospective cohort study of 88,905 UK Biobank participants, individuals in the highest category of nighttime light exposure (91st–100th percentile) had a 45% higher risk of HF incidence compared with those in the darkest nighttime environments (HR 1.45, 95% CI 1.24–1.69) [98]. Although direct evidence linking light pollution specifically to HF remains limited, growing mechanistic and population‑level data support public health and clinical strategies to reduce excessive night-time light exposure and to separate ALAN from urban co-exposures such as traffic noise and air pollution. Interestingly, one study linking ALAN to HF showed that there was a larger-magnitude association in the younger sample population, although the mean age of study participants was 62.4 years old, implying that the population was already ageing and other confounders might have been at play [98].

### Social Environment

Social determinants such as socioeconomic status (SES), social deprivation, living conditions, and access to healthcare, as well as psychosocial factors such as social isolation and loneliness, have been consistently associated with cardiovascular risk and a higher HF incidence, increased hospitalization rates, and mortality [99–103]. Recent evidence further indicates that both individual-level SES indicators (e.g., income, education, and employment) and area-level measures of social deprivation are associated with adverse HF outcomes. A systematic review reported that low SES was associated with higher HF incidence in 72% of studies, higher HF prevalence in 75% of studies, and increased mortality in 45% of studies, with reported hazard ratios ranging from 1.12 to 2.58 for HF incidence and from 1.19 to 1.98 for mortality depending on the SES indicator used [104]. Likewise, a nationwide U.S. analysis of 1,905 counties demonstrated that risk-adjusted HF hospitalization rates increased by 2.5 per 100,000 population for every 0.01-unit increase in the Social Vulnerability Index (SVI) and by 2.7 per 100,000 population for every 1-unit increase in the Social Deprivation Index (SDI) [105]. At the same time, individuals living in disadvantaged environments are often exposed to additional environmental stressors, including air pollution, traffic noise, and limited access to green space, which may further increase cardiovascular risk and HF [8, 9]. For instance, social isolation and loneliness are highly prevalent in elderly populations and have been associated with adverse cardiovascular outcomes. Several large cohort studies suggest that both factors are linked to an increased risk of incident HF as well as poorer outcomes in patients with established HF [101, 106]. From a mechanistic perspective, social isolation and loneliness are associated with activation of the SNS and the HPA axis, resulting in elevated stress hormone levels, systemic inflammation, and endothelial dysfunction [100, 107]. These responses overlap with pathways described for other environmental exposures and may contribute to cardiac remodelling and progression of HF [9]. In addition, psychosocial factors such as depression and chronic stress are common in HF patients. These conditions are associated with reduced adherence to medical therapy, unhealthy lifestyle patterns, and delayed healthcare utilization, all of which may negatively affect clinical outcomes [108, 109]. Importantly, social and environmental disadvantages rarely occur in isolation but tend to cluster, thereby further increasing vulnerability in older HF patients with multimorbidity [28, 29].

### Other Determinants

#### Green Space and Walkable Neighbourhoods

Greener, more walkable neighbourhoods appear to protect against the development of HF, mainly by improving upstream cardiovascular risk factors. Living in areas with more residential greenness is associated with modestly lower incidence of HF and cardiovascular disease, whereas effects on outcomes after HF onset are less clear [15, 110]. Higher neighbourhood walkability is associated with lower rates of obesity, diabetes, and hypertension, and fewer cardiovascular events, suggesting that supportive environments for walking and access to green space help reduce HF risk by improving cardiometabolic health [111, 112]. In a large population-based cohort study of nearly 1.3 million adults in Canada, each 1-unit increase in residential greenness (NDVI) was associated with a 6% lower risk of incident HF (HR 0.94, 95% CI 0.93–0.96) [110]. Similarly, Bober et al. analyzed 756 recently hospitalised patients with HF with reduced ejection fraction (HFrEF) (mean age 64 years). Patients living in the least walkable neighbourhoods had a significantly higher risk of 12-month all-cause mortality compared with those living in more walkable areas (HR 1.70, 95% CI 1.11–2.61), whereas residents of the most deprived neighbourhoods had a higher risk of cardiovascular-related readmission within 12 months (HR 1.39, 95% CI 1.09–1.78) [113]. There is a lack of direct investigation on the positive effects of greenness and walkability on HF, but the improvements in underlying cardiometabolic health support the connection.

#### Exercise

Regular exercise substantially lowers the risk of developing HF and improves survival, symptoms, and exercise capacity in patients with established HF, while a sedentary lifestyle increases both HF incidence and mortality [114, 115]. Large cohorts show that meeting guideline-level physical activity is associated with lower HF risk, and randomized trials and cardiac rehabilitation studies demonstrate that structured training reduces HF hospitalizations and all‑cause mortality [116–118]. In a prospective cohort study of nearly 95,000 UK Biobank participants (mean age approximately 56 years), device-measured physical activity was strongly associated with a lower incidence of HF. The participants performing 150–300 min/week of moderate-intensity physical activity had a 63% lower HF risk (HR 0.37, 95% CI 0.34–0.41), while those performing 75–150 min/week of vigorous-intensity activity had a 66% lower risk (HR 0.34, 95% CI 0.25–0.46) compared with inactive individuals [116]. In the ARIC study, which followed 13,810 middle-aged adults (mean age 55 years) without HF at baseline for a median of 26 years, participants meeting recommended physical activity levels had a 22% lower risk of developing HF than inactive individuals (HR 0.78, 95% CI 0.72–0.85) [119]. Similar protective associations were observed among participants with hypertension, obesity, diabetes, and metabolic syndrome, whereas no significant reduction in HF risk was observed among individuals with established atherosclerotic cardiovascular disease. Regular exercise is especially important in elderly HF patients, with studies showing that daily physical activity is strongly and inversely associated with mortality, even after accounting for comorbidities and disability [120, 121].

#### Diet

Diet affects both the risk of developing HF and outcomes in established disease. Heart‑healthy patterns such as the Mediterranean and DASH diets, rich in fruits, vegetables, whole grains, legumes, nuts, and unsaturated fats, are associated with lower HF incidence and mortality [122, 123], in part through improved blood pressure and lipid profiles and reduced inflammation [124]. Supporting this, Lara et al. followed 16,068 US adults (mean age 64 years) for a median of 8.7 years and found that participants with the highest adherence to a plant-based dietary pattern had a 41% lower risk of incident HF than those with the lowest adherence (HR 0.59, 95% CI 0.41–0.86), whereas a Southern dietary pattern was associated with a higher HF risk (HR 1.72, 95% CI 1.20–2.46) [122]. In patients with established HF, Joseph et al. analysed 3,798 participants from 25 countries enrolled in the G-CHF registry and observed that higher legume intake was associated with a lower risk of death or HF hospitalization (HR 0.80, 95% CI 0.65–0.98 for ≥ 0.5 servings/day vs. < 0.1 servings/day), whereas higher refined grain intake was associated with an increased risk of HF hospitalization (HR 1.76, 95% CI 1.30–2.39 for > 3 servings/day vs. < 1 serving/day) [32]. Moderate vegetable intake was also associated with a lower risk of HF hospitalization (HR 0.77, 95% CI 0.61–0.97). In people with HF, excess sodium promotes fluid retention and decompensation, while malnutrition and cardiac cachexia are common in advanced stages and worsen prognosis, making individualized nutritional support (adequate energy and protein, careful sodium and fluid management) an important component of HF care [125, 126]. In elderly HF cohorts, malnutrition and cardiac cachexia are very common and older HF patients with poor nutritional status have markedly higher mortality, more hospitalizations, and worse quality of life than well‑nourished peers [127, 128]. Elderly HF patients generally have lower intakes of key macro‑ and micronutrients than age‑matched controls [129], underscoring the importance of adequate nutrition in HF management.

## Mechanistic Intersection Between Aging, HF and Exposomic Determinants

**Fig. 4:**
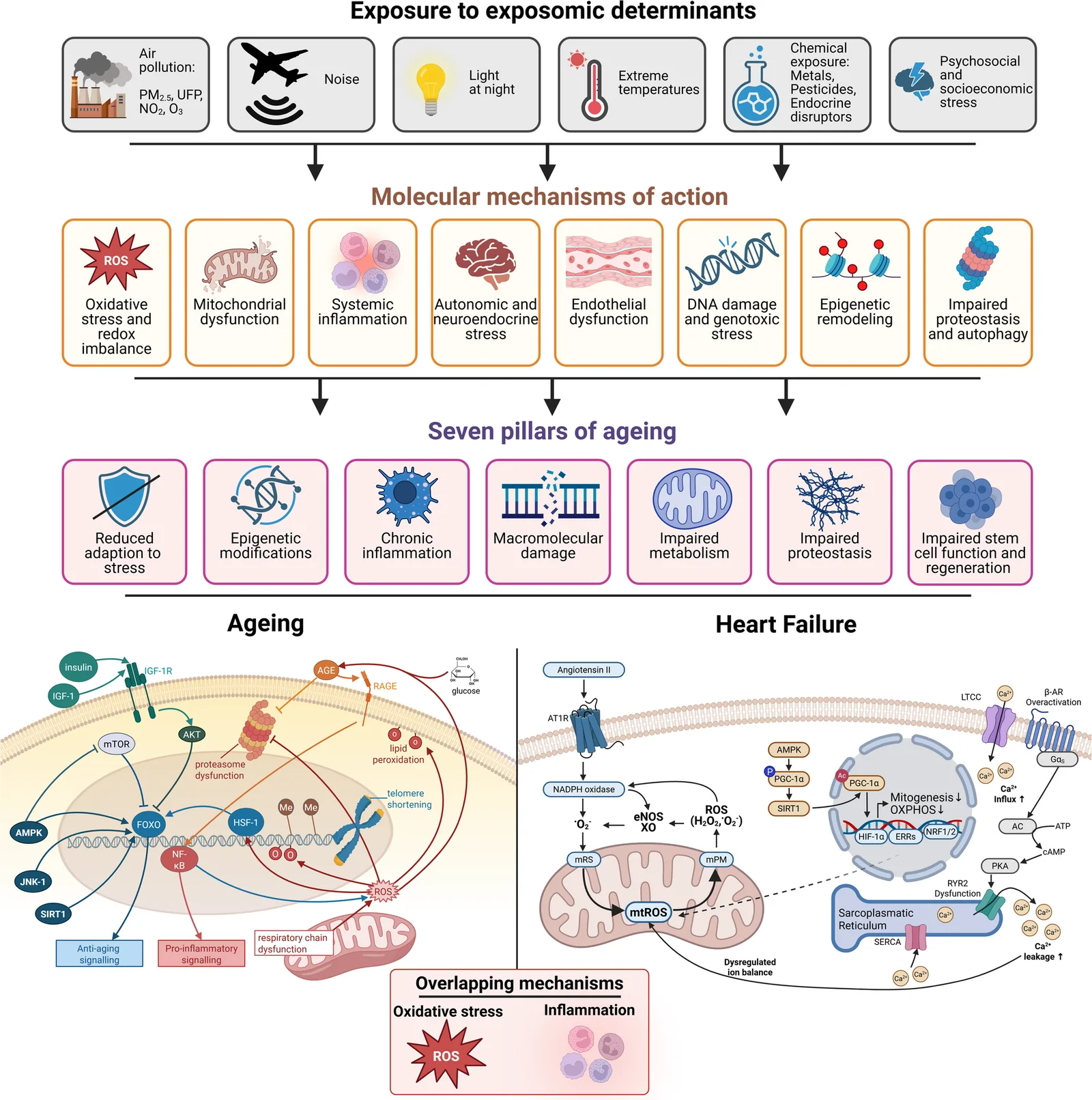
Overview of the mechanisms by which exposomic determinants intersect with molecular mechanisms of ageing. The upper panel shows how the exposomic risk factors influence the seven pillars of ageing. The bottom panel shows mechanistic similarities in ageing and in heart failure. Adapted from [162] (CC BY-NC-ND 4.0) [27], and [163] (CC BY 4.0 licence), with permission. Created with BioRender.com

Aging, heart failure, and the exposome are linked by converging biochemical pathways centered on oxidative stress, chronic inflammation, impaired proteostasis/mitophagy, neurohormonal activation, and maladaptive remodeling, with environmental exposures accelerating aging hallmarks in the cardiovascular system and thereby predisposing to and worsening heart failure [9, 131], which shares similar pathomechanisms [27]. The overview of the mechanistic pathways shared by aging, HF and environmental determinants is shown in Fig. 4.

### Oxidative Stress and Mitochondrial Dysfunction

Excessive reactive oxygen species (ROS) production from mitochondrial ETC leakage or enzymatic activity (e.g. NADPH oxidases) damages mitochondrial DNA, lipids, and proteins, impairs oxidative phosphorylation, and disrupts mitochondrial dynamics [132]. This activates redox‑sensitive signalling through NF‑κB, NLRP3, and MAPKs, decreases ·NO bioavailability, and promotes cardiomyocyte apoptosis, necrosis, and fibrosis [133]. Oxidative stress is a primary hallmark of cardiovascular aging, driving progressive myocardial fibrosis, diastolic dysfunction, and ventricular stiffening [134]. Aging hearts also show impaired antioxidant defences like reduction in Nrf2 transcription and SOD activity, so the same ROS burden causes more structural damage and HF susceptibility [132, 135].

Outdoor/indoor PM_2.5_ and ultrafine particles, NO_2_, ozone, and metal‑laden particles generate ROS directly and via surface‑bound PAHs and metals, triggering mitochondrial damage and oxLDL formation. Noise, heat, and other environmental stressors also promote systemic oxidative stress through neurohormonal activation and impaired circadian regulation [136].

### Inflammation and “Inflammaging”

Aging is associated with chronic activation of NF‑κB, NLRP3 inflammasome, and TLR pathways, which increases TNF‑α, IL‑1β, IL‑6, and adhesion molecules, driving endothelial activation and leukocyte recruitment. ROS and DAMPs from injured cells sustain this loop, promoting LDL oxidation, foam cell formation, matrix metalloproteinase activity, and extracellular matrix remodelling [137]. Inflammaging is an integrative hallmark of cardiovascular aging, linked to arterial stiffness, endothelial dysfunction, and atherosclerotic progression [138]. In the aged heart, low‑grade chronic inflammation promotes interstitial fibrosis and impairs both systolic and diastolic function, lowering the threshold for HF [139, 140].

PM_2.5_ triggers pulmonary cytokine production and bone‑marrow monocyte mobilization, enhancing plaque inflammation and vulnerability. Noise, ALAN, and psychosocial stress increase sympathetic tone and HPA activation, fostering systemic inflammatory states. Temperature extremes and some toxic chemicals (e.g. metals) also induce inflammatory and endothelial activation pathways [9, 12, 136].

### Impaired Autophagy and Proteostasis

In aged cells macroautophagy and mitophagy are reduced due to AMPK deactivation and mTORC1 overactivity which limits clearance of damaged proteins and organelles. This leads to proteotoxic stress, accumulation of dysfunctional mitochondria, and activation of cell‑death and senescence pathways in cardiomyocytes [141]. Disabled macroautophagy and loss of proteostasis are primary hallmarks of cardiovascular aging and are strongly linked to age‑related cardiomyopathy, hypertrophy, and contractile dysfunction [142]. Aging‑related decline of AMPK–SIRT1 signalling exacerbates fibrosis and pump dysfunction, especially under hemodynamic or ischemic stress, promoting HF [132].

Chronic ROS and inflammatory signalling from air pollution and other exposures overload autophagy pathways and further impair lysosomal and mitophagic flux. Certain toxicants (e.g. metals, some organic pollutants) directly interfere with proteostasis and mitochondrial quality control, aggravating these defects [136].

### Genomic Instability and Epigenetic Alterations

Oxidative stress causes DNA damage and telomere shortening, while maladaptive DNA repair and clonal hematopoiesis promote pro‑inflammatory hematopoietic clones. Epigenetic changes, including DNA methylation, histone marks, and non‑coding RNAs, reprogram expression of stress‑response, inflammatory, fibrotic, and metabolic genes, driving the vicious cycle [143]. Genomic instability and telomere dysfunction are central aging hallmarks, contributing to cellular senescence in vascular and cardiac cells and to pro‑inflammatory hematopoietic profiles [144]. Age‑related epigenetic drift reshapes cardiovascular gene networks, creating a predisposition to HF alongside other risk factors [132, 141].

Air pollution, metals, and other environmental toxicants induce DNA damage and alter DNA methylation patterns in cardiovascular and immune cells. Chronic environmental stressors (noise, light, social/urban stress) are linked to epigenetic changes in stress‑axis and inflammatory pathways from early life [9, 136].

### Cellular Senescence

Persistent DNA damage and oxidative stress trigger the p53/p21 and p16INK4a pathways, leading to irreversible growth arrest and the acquisition of a pro-inflammatory senescence-associated secretory phenotype (SASP; including cytokines, growth factors, and proteases) [145, 146]. Senescent endothelial cells, fibroblasts, and cardiomyocytes secrete factors that alter the extracellular matrix, promote fibrosis, and impair contractility and vascular tone [147, 148]. Senescence is a core hallmark of cardiovascular aging, driving stiffness, impaired vasodilation, and maladaptive remodelling [149, 150]. Senescent cell accumulation in the aged myocardium and vasculature contributes to HF with preserved and reduced ejection fraction (HFpEF/HFrEF) [149, 151, 152].

Repeated ROS and inflammatory induction by PM_2.5_, combustion products, and environmental toxicants accelerate the initiation of senescence in vascular and cardiac cells [153]. Chronic psychosocial and circadian stress (noise, light at night, shift‑like patterns) likely enhance senescence via hormonal and metabolic dysregulation [154, 155].

### Dysregulated Neurohormonal and Circadian Signalin

Activation of sympathetic nervous system and rennin-angiotensin-aldosterone system (RAAS) increases catecholamines, angiotensin II, aldosterone, and cortisol, promoting vasoconstriction, hypertrophy, and fibrosis via angiotensin II type 1 receptor (AT1R), mineralocorticoid receptors, and downstream kinases (e.g. ROCK) [132, 136]. Circadian disruption alters clock‑gene control of metabolism, inflammation, and vascular tone, uncoupling protective day–night rhythms [136]. Aging is associated with heightened basal sympathetic tone, altered baroreflexes, and RAAS dysregulation, contributing to hypertension, LV hypertrophy, and HF [9]. Dampened circadian amplitude in older adults aggravates metabolic and inflammatory disturbances that predispose to HF [156, 157].

Traffic noise, night‑time light, and other urban stressors chronically activate stress-hormone signalling, disrupt sleep, and blunt circadian rhythms [154]. The impact of noise on hypertensive patients was already shown in the year 1977 [158]. Heat waves and cold exposure trigger adrenergic and RAAS responses, raising blood pressure and cardiac workload, especially in vulnerable older individuals [51].

### Fibrosis and Maladaptive Structural Remodelling

Transforming growth factor-beta (TGF‑β), angiotensin II, inflammatory cytokines, and ROS activate fibroblasts, increase collagen I/III deposition, and alter MMP/TIMP balance, leading to extracellular matrix accumulation and stiffness [132, 137]. Microvascular rarefaction and endothelial dysfunction further promote hypoxia and fibrosis, feeding back to worsen contractile dysfunction [132, 159]. Myocardial and vascular fibrosis are major structural expressions of cardiovascular aging, underpinning diastolic dysfunction, HFpEF, and increased HF risk [160]. Age‑associated inflammaging and oxidative stress amplify fibrotic signalling, making older hearts more prone to maladaptive remodelling in response to any additional stressor [161].

Air pollution–driven oxidative stress and inflammation enhance fibrotic pathways in myocardium and vessels, especially when combined with hypertension or ischemia [12]. Environmental stressors, such as noise and extreme temperatures, increase blood pressure, afterload, or ischemic burden, and indirectly promote fibrotic remodelling and HF progression [9].

## What Can We Learn from Non-Mammalian Exposure Models?

Zebrafish (*Danio rerio*) has emerged as an excellent non-mammalian model organism for the study of aging and HF due to its approximately 70% homology with human protein-coding genes and approximately 84% of genes associated with human diseases have Zebra fish orthologs. The advantages it offers as a model organism like having transparent embryos, short life cycle are beneficial for cardiac aging and HF studies. Research using *Danio rerio* have yielded translationally relevant findings in some core areas like cellular senescence, structural remodeling, loss of the cardiac regeneration reserve, and toxicant-induced cardiac anomalies [164]. Progressive accumulation of β-galactosidase (SA-β-gal) in aging tissues and accelerating senescence by shortening of telomeres, is considered a key aging mechanism. Genetic loss of telomerase demonstrated a link between telomere shortening and induction of a p53–mTOR/AKT-mediated switch from apoptosis to senescence and the suppression of mitochondrial antioxidant SOD2, directly connecting telomere attrition to oxidative stress induced in cardiac-tissues [165]. In addition, telomerase depletion was found to upregulate senescence markers, neuroinflammation, and blood–brain barrier disruption in non-canonical tissues, demonstrating potential non-canonical functions of telomerase in cardiac homeostasis during aging. Use of *Danio rerio* in cardiac structural remodeling shows age dependence and closely reflects similarities with human cardiac disease [166]. Progressive development of cardiomyocyte hypertrophy, ventricular fibrosis and valvular lesions have been documented with fish age. Slightly older zebrafish (> 2.5 years) have been shown to develop spontaneous valvular disease, increased valve volume and enhanced immune-cell infiltration, alongside echocardiographic dysfunction similar to human valvular disease [167]. The remarkable ability of zebrafish heart to be studied for regenerative capacity was established by the finding that post-cryoinjury, young fish could regenerate the ventricular myocardium within approximately 90 days by cardiomyocyte dedifferentiation and proliferation [168]. This expanded its potential as a model to study heart regeneration that mammals lack. This regenerative capacity is observed to decrease with age as older fish have impaired and variable regeneration which is associated with macrophage accumulation, linking inflammaging to the failure of regeneration [169]. A progressive fibrosis, decreased swimming capacity, increased levels of cardiac insufficiency markers, loss of the proliferative cardiomyocyte pool, and lifetime exercise enhanced fitness could not restore proliferative capacity of cardiomyocytes. *Danio rerio* exposure models have proved to mimic clinically relevant cardiotoxic mechanisms. Exposure to metabolic wastes in aqueous media activates cardiac dysfunction which is also seen in chronic kidney disease patients [170]. Further, exposures to common environmental toxicants, such as glyphosate, cause cardiomyocyte senescence and inhibit proliferation, thereby accelerating cardiac myopathy. When combined with the conservation of aging pathway, transparent phenotyping, and scalable exposures, Zebra fish provides a high-throughput link between *in vitro* screening and mammalian models for aging and HF.

*Drosophila melanogaster* shares conserved homology of diseased genes up to 65% to 70% with humans. This makes Drosophila an ideal model to study various metabolic disorders. It includes (1) a short life span and short developmental cycle, (2) easy genetic manipulation, (3) low maintenance cost in comparison with other models. It is widely used to study the many metabolic defects, including aging and cardiac dysfunctions [171, 172]. The genes that regulate aging are conserved between Drosophila and humans. Evolutionarily conserved pathways of IGF-1 (InR), TOR and JNK, which govern longevity, are present in Drosophila [173]. The epigenetic alterations during aging have been studied in Drosophila [174]. The misexpression, expression and overexpression of genes like Sirtuins and dFoxo, which are key regulators of the aging pathway, can be easily monitored due to their simpler physiological systems [175]. Drosophila is a good model to study cardiac dysfunction. The genetic network that regulates cardiogenesis is conserved between Drosophila and mammals [171, 176]. Like mammals, an HFD also causes lipid accumulation and heart dysfunction in Drosophila [171]. Like in vertebrates, hypoxic conditions alter the HIF-α expression level and the functioning of the heart of Drosophila [177]. The rhythmic contraction of the heart is maintained by actin and myosin proteins in both mammals and Drosophila [178]. The human gene NKX2-5 is conserved in Drosophila as Tinman, a key regulator of heart development [179] Wnt, Notch, BMP and Hippo signaling play a key role in the structural and functional maintenance of the heart in both organisms [180]. Therefore, Drosophila may represent a promising model for conducting high-throughput studies on the impact of environmental exposures on HF during the aging process.

## Conclusion

A growing body of evidence supports the view that HF in older adults cannot be understood solely through traditional risk factors and cardiac intrinsic pathology but instead emerges from the dynamic interplay between biological aging and the totality of environmental and behavioural exposures across the life course. Taken together, the data reviewed here indicate that age‑related alterations in stress adaptation, epigenetic regulation, inflammation, macromolecular integrity, metabolism, proteostasis, and regenerative capacity render the myocardium and vasculature exquisitely sensitive to external stressors. In this context, the exposome acts not merely as a trigger of acute decompensations, but also as a chronic modulator and accelerator of the very biochemical processes that underpin cardiovascular aging and HF. Conceptually, this review argues for a shift from a narrow, organ‑centred view of HF in older adults toward an integrative, life‑course and systems‑level perspective. Clinically, it underscores the need to recognize exposomic determinants as modifiable contributors to risk and progression of HF. From a public health and policy standpoint, interventions that reduce ambient pollution, noise, light at night, thermal stress, and social deprivation, while promoting green space, walkability, physical activity, and healthy diets, should be considered part of a comprehensive strategy for HF prevention and management in aging societies. Future research should move beyond isolated exposures and single‑pathway analyses toward multi‑omics, longitudinal, and intervention studies that capture the complexity of exposome–aging–HF interactions, identify high‑risk phenotypes, and test whether targeted modification of environmental and behavioural determinants can meaningfully alter HF incidence, progression, and outcomes in older populations.

## Key References

- Hahad O, Wass S, Rajagopalan S, Abohashem S, Hao H, Navas-Acien A, Bellumkonda L, Chen K, Brook RD, Nasir K, Lurz P, Lanfear DE, Bhimaraj A, Al-Kindi S. The environmental exposome in heart failure risk and progression. Nat Rev Cardiol. 2026 Jul;23(7):457–472. https://doi.org/10.1038/s41569-026-01247-1.
  - ○ State of the art review on the current understanding of the involvement of exposomic risk factors on heart failure.
- Wild CP. Complementing the genome with an “exposome”: the outstanding challenge of environmental exposure measurement in molecular epidemiology. Cancer Epidemiol Biomarkers Prev. 2005 Aug;14(8):1847-50. https://doi.org/10.1158/1055-9965.EPI-05-0456.
  - ○ First publication coining the term exposome and identifying its importance.
- Münzel T, Sørensen M, Lelieveld J, Landrigan PJ, Kuntic M, Nieuwenhuijsen M, Miller MR, Schneider A, Daiber A. A comprehensive review/expert statement on environmental risk factors of cardiovascular disease. Cardiovasc Res. 2025 Sep 29;121(11):1653–1678. https://doi.org/10.1093/cvr/cvaf119.
  - ○ An expert statement on the necessity for implementation of environmental risk factors in the cardiovascular disease mitigation guidelines.
- Abdellatif M, Rainer PP, Sedej S, Kroemer G. Hallmarks of cardiovascular ageing. Nat Rev Cardiol. 2023 Nov;20(11):754–777. https://doi.org/10.1038/s41569-023-00881-3.
  - ○ State of the art review that provides an overview on molecular mechanisms of cardiovascular ageing.

## Data Availability

No datasets were generated or analysed during the current study.

## References

[1] Clinic M. Heart failure*.* 2026 [cited 2026 20.04.2026]. Available from: https://www.mayoclinic.org/diseases-conditions/heart-failure/symptoms-causes/syc-20373142

[2] Association AH. What is Heart Failure? 2026 [cited 2026 20.04.2026]. Available from: https://www.heart.org/en/health-topics/heart-failure/what-is-heart-failure

[3] Shams P, Malik A, Chhabra L. Heart Failure (Congestive Heart Failure), in StatPearls. 2026; Treasure Island (FL).

[4] Wild CP. Complementing the genome with an exposome: the outstanding challenge of environmental exposure measurement in molecular epidemiology. Cancer Epidemiol Biomarkers Prev. 2005;14(8):1847–50. 10.1158/1055-9965.EPI-05-0456 16103423

[5] Ndlovu N, Nkeh-Chungag BN. Impact of Indoor Air Pollutants on the Cardiovascular Health Outcomes of Older Adults: Systematic Review. Clin Interv Aging. 2024;19:1629–39. 10.2147/CIA.S480054 PMC11453128 39372166

[6] Lim YH, et al. Long-Term Exposure to Air Pollution, Road Traffic Noise, and Heart Failure Incidence: The Danish Nurse Cohort. J Am Heart Assoc. 2021;10(20):e021436. 10.1161/JAHA.121.021436 PMC8751865 34612059

[7] Escolar V, et al. Impact of environmental factors on heart failure decompensations. ESC Heart Fail. 2019;6(6):1226–32. 10.1002/ehf2.12506 PMC6989274 31483570

[8] Munzel T, et al. The contribution of the exposome to the burden of cardiovascular disease. Nat Rev Cardiol. 2023;20(10):651–69. 10.1038/s41569-023-00873-3 37165157

[9] Hahad O, et al. The environmental exposome in heart failure risk and progression. Nat Rev Cardiol; 2026. 10.1038/s41569-026-01247-1 41582253

[10] Schwinger RHG. Pathophysiology of heart failure. Cardiovasc Diagn Ther. 2021;11(1):263–76. 10.21037/cdt-20-302 PMC7944197 33708498

[11] Kennedy BK, et al. Geroscience: linking aging to chronic disease. Cell. 2014;159(4):709–13. 10.1016/j.cell.2014.10.039 PMC4852871 25417146

[12] Munzel T, et al. A comprehensive review/expert statement on environmental risk factors of cardiovascular disease. Cardiovasc Res. 2025;121(11):1653–78. 10.1093/cvr/cvaf119 PMC12477681 40795898

[13] Osborne MT, et al. A neurobiological mechanism linking transportation noise to cardiovascular disease in humans. Eur Heart J. 2020;41(6):772–82. 10.1093/eurheartj/ehz820 PMC7006229 31769799

[14] Munzel T, et al. Cardiovascular effects of environmental noise exposure. Eur Heart J. 2014;35(13):829–36. 10.1093/eurheartj/ehu030 PMC3971384 24616334

[15] James P, et al. Assessing greenspace and cardiovascular disease risk through deep learning analysis of street-view imagery in the US-based nationwide Nurses’ Health Study. Environ Epidemiol. 2026;10(1):e442. 10.1097/EE9.0000000000000442 PMC12818865 41567830

[16] Miller JG, et al. Air pollution is associated with elevated HPA-Axis response to stress in anxious adolescent girls. Compr Psychoneuroendocrinol. 2020;4:100015. 10.1016/j.cpnec.2020.100015 PMC9216601 35755623

[17] Taylor-Clark TE. Air Pollution-Induced Autonomic Modulation. Physiol (Bethesda). 2020;35(6):363–74. 10.1152/physiol.00017.2020 PMC7864236 33052776

[18] Molcan L, et al. Artificial light at night suppresses the day-night cardiovascular variability: evidence from humans and rats. Pflugers Arch. 2024;476(3):295–306. 10.1007/s00424-023-02901-0 PMC10847188 38177874

[19] Scata C et al. Social Isolation: A Narrative Review on the Dangerous Liaison between the Autonomic Nervous System and Inflammation. Life (Basel). 2023;13(6). 10.3390/life13061229 PMC10301441 37374012

[20] Dimitrov-Discher A, et al. Residential green space and air pollution are associated with brain activation in a social-stress paradigm. Sci Rep. 2022;12(1):10614. 10.1038/s41598-022-14659-z PMC9226020 35739150

[21] Munzel T, et al. Soil and water pollution and cardiovascular disease. Nat Rev Cardiol. 2025;22(2):71–89. 10.1038/s41569-024-01068-0 39317838

[22] Kim HJ, Hwang J, Park JH. Long-Term Exposure to Ambient Air Pollution and Metabolic Syndrome and Its Components. J Obes Metab Syndr. 2025;34(2):91–104. 10.7570/jomes24036 PMC12067007 40090381

[23] Khalil WJ et al. Environmental Pollution and the Risk of Developing Metabolic Disorders: Obesity and Diabetes. Int J Mol Sci. 2023;24(10). 10.3390/ijms24108870 PMC10219141 37240215

[24] Arneth B. Interactions among nutrition, metabolism and the immune system in the context of starvation and nutrition-stimulated obesity. Nutr Diabetes. 2025;15(1):26. 10.1038/s41387-025-00383-w PMC12152128 40494849

[25] Thacher JD, et al. Long-Term Exposure to Transportation Noise and Risk for Type 2 Diabetes in a Nationwide Cohort Study from Denmark. Environ Health Perspect. 2021;129(12):127003. 10.1289/EHP9146 PMC8638828 34855467

[26] Tracy E, Rowe G, LeBlanc AJ. Cardiac tissue remodeling in healthy aging: the road to pathology. Am J Physiol Cell Physiol. 2020;319(1):C166–82. 10.1152/ajpcell.00021.2020 PMC7468888 32432929

[27] Munzel T, et al. Pathophysiological role of oxidative stress in systolic and diastolic heart failure and its therapeutic implications. Eur Heart J. 2015;36(38):2555–64. 10.1093/eurheartj/ehv305 PMC7959410 26142467

[28] Scholten M, et al. Comorbidities in heart failure patients that predict cardiovascular readmissions within 100 days-An observational study. PLoS ONE. 2024;19(1):e0296527. 10.1371/journal.pone.0296527 PMC10760770 38165943

[29] Sorop O, et al. Multiple common comorbidities produce left ventricular diastolic dysfunction associated with coronary microvascular dysfunction, oxidative stress, and myocardial stiffening. Cardiovasc Res. 2018;114(7):954–64. 10.1093/cvr/cvy038 PMC5967461 29432575

[30] Wagner JUG, et al. Aging impairs the neurovascular interface in the heart. Science. 2023;381(6660):897–906. 10.1126/science.ade4961 37616346

[31] Meta-analysis Global Group in Chronic, Heart F. The survival of patients with heart failure with preserved or reduced left ventricular ejection fraction: an individual patient data meta-analysis. Eur Heart J. 2012;33(14):1750–7. 10.1093/eurheartj/ehr254 21821849

[32] Joseph P, et al. Diet and Clinical Outcomes in a Heart Failure Population. JACC Heart Fail. 2026;14(4):102728. 10.1016/j.jchf.2025.102728 41165628

[33] Shah AS, et al. Global association of air pollution and heart failure: a systematic review and meta-analysis. Lancet. 2013;382(9897):1039–48. 10.1016/S0140-6736(13)60898-3 PMC3809511 23849322

[34] Jia Y, et al. Effect of Air Pollution on Heart Failure: Systematic Review and Meta-Analysis. Environ Health Perspect. 2023;131(7):76001. 10.1289/EHP11506 PMC10317211 37399145

[35] Wang M, et al. Joint exposure to various ambient air pollutants and incident heart failure: a prospective analysis in UK Biobank. Eur Heart J. 2021;42(16):1582–91. 10.1093/eurheartj/ehaa1031 PMC8060055 33527989

[36] Bai L, et al. Exposure to ambient air pollution and the incidence of congestive heart failure and acute myocardial infarction: A population-based study of 5.1 million Canadian adults living in Ontario. Environ Int. 2019;132:105004. 10.1016/j.envint.2019.105004 31387019

[37] Ward-Caviness CK, et al. Long-Term Exposure to Particulate Air Pollution Is Associated With 30-Day Readmissions and Hospital Visits Among Patients With Heart Failure. J Am Heart Assoc. 2021;10(10):e019430. 10.1161/JAHA.120.019430 PMC8200693 33942627

[38] Mentias A, et al. Ambient Air Pollution Exposure and Adverse Outcomes Among Medicare Beneficiaries With Heart Failure. J Am Heart Assoc. 2024;13(15):e032902. 10.1161/JAHA.123.032902 PMC11964021 39082400

[39] Al-Kindi SG, et al. Ambient Air Pollution and Mortality After Cardiac Transplantation. J Am Coll Cardiol. 2019;74(24):3026–35. 10.1016/j.jacc.2019.09.066 31865970

[40] Vieira JL, et al. Respiratory Filter Reduces the Cardiovascular Effects Associated With Diesel Exhaust Exposure: A Randomized, Prospective, Double-Blind, Controlled Study of Heart Failure: The FILTER-HF Trial. JACC Heart Fail. 2016;4(1):55–64. 10.1016/j.jchf.2015.07.018 26738952

[41] Humphrey JL, et al. Disentangling impacts of multiple pollutants on acute cardiovascular events in New York city: A case-crossover analysis. Environ Res. 2024;242:117758. 10.1016/j.envres.2023.117758 PMC11378578 38029813

[42] Shi Y, et al. Association between long-term exposure to ambient air pollution and clinical outcomes among patients with heart failure: Findings from the China PEACE Prospective Heart Failure Study. Ecotoxicol Environ Saf. 2021;222:112517. 10.1016/j.ecoenv.2021.112517 34303044

[43] Weldy CS, et al. In utero exposure to diesel exhaust air pollution promotes adverse intrauterine conditions, resulting in weight gain, altered blood pressure, and increased susceptibility to heart failure in adult mice. PLoS ONE. 2014;9(2):e88582. 10.1371/journal.pone.0088582 PMC3922927 24533117

[44] Carll AP, et al. Particulate matter inhalation exacerbates cardiopulmonary injury in a rat model of isoproterenol-induced cardiomyopathy. Inhal Toxicol. 2010;22(5):355–68. 10.3109/08958370903365692 20121584

[45] Liu M, et al. Association between road traffic noise exposure and heart failure: A systematic review and meta-analysis of prospective cohort studies. Public Health. 2025;241:107–14. 10.1016/j.puhe.2025.01.029 39970506

[46] Yang T, et al. Long-Term Exposure to Road Traffic Noise and Incident Heart Failure: Evidence From UK Biobank. JACC Heart Fail. 2023;11(8 Pt 1):986–96. 10.1016/j.jchf.2023.04.003 37227391

[47] Seidler A, et al. Aircraft, road and railway traffic noise as risk factors for heart failure and hypertensive heart disease-A case-control study based on secondary data. Int J Hyg Environ Health. 2016;219(8):749–58. 10.1016/j.ijheh.2016.09.012 27667192

[48] Bai L, et al. Exposure to Road Traffic Noise and Incidence of Acute Myocardial Infarction and Congestive Heart Failure: A Population-Based Cohort Study in Toronto, Canada. Environ Health Perspect. 2020;128(8):87001. 10.1289/EHP5809 PMC7422718 32783534

[49] Thacher JD, et al. Exposure to transportation noise and risk for cardiovascular disease in a nationwide cohort study from Denmark. Environ Res. 2022;211:113106. 10.1016/j.envres.2022.113106 35304113

[50] Zhang L, Han X. Relationship between Noise Exposure and Prognosis of Patients with Heart Failure: A Retrospective Analysis. Noise Health. 2024;26(120):51–7. 10.4103/nah.nah_101_23 PMC11141694 38570311

[51] Alahmad B, et al. Associations Between Extreme Temperatures and Cardiovascular Cause-Specific Mortality: Results From 27 Countries. Circulation. 2023;147(1):35–46. 10.1161/CIRCULATIONAHA.122.061832 PMC9794133 36503273

[52] Hanneman K et al. Nonoptimal Temperature and Cardiovascular Health: A Scientific Statement From the American Heart Association. Circulation. 2026. 10.1161/CIR.0000000000001419 41884891

[53] Yu X et al. Nonlinear Relation Between Cardiac Mortality and Excess Temperature in Heatwaves: Exposure Response in 2.39 Million Patients. J Am Coll Cardiol, 2025. 10.1016/j.jacc.2025.01.034 40131259

[54] Meade RD, et al. Physiological responses to 9 hours of heat exposure in young and older adults. Part I: Body temperature and hemodynamic regulation. J Appl Physiol (1985). 2023;135(3):673–87. 10.1152/japplphysiol.00227.2023 37439239

[55] Meade RD, et al. Meta-analysis of heat-induced changes in cardiac function from over 400 laboratory-based heat exposure studies. Nat Commun. 2025;16(1):2543. 10.1038/s41467-025-57868-6 PMC11909281 40087302

[56] Cui J, et al. Effects of heat stress on thermoregulatory responses in congestive heart failure patients. Circulation. 2005;112(15):2286–92. 10.1161/CIRCULATIONAHA.105.540773 16216975

[57] Munzel T, et al. Challenges posed by climate hazards to cardiovascular health and cardiac intensive care: implications for mitigation and adaptation. Eur Heart J Acute Cardiovasc Care. 2024;13(10):731–44. 10.1093/ehjacc/zuae113 PMC11518858 39468673

[58] Khatana SAM, et al. Projected Change in the Burden of Excess Cardiovascular Deaths Associated With Extreme Heat by Midcentury (2036–2065) in the Contiguous United States. Circulation. 2023;148(20):1559–69. 10.1161/CIRCULATIONAHA.123.066017 PMC10840949 37901952

[59] Hundessa S, et al. Global and Regional Cardiovascular Mortality Attributable to Nonoptimal Temperatures Over Time. J Am Coll Cardiol. 2024;83(23):2276–87. 10.1016/j.jacc.2024.03.425 38839202

[60] Ni W, et al. Short-Term Exposure to Low and High Temperatures and Mortality Among Patients With Heart Failure in Sweden. JAMA Cardiol. 2025;10(12):1276–84. 10.1001/jamacardio.2025.3932 PMC12547678 41123904

[61] Carrete A, Codina P, Bayes-Genis A. Influence of temperature on haemodynamic changes in heart failure: A CardioMEMS pulmonary artery monitoring analysis. Eur J Heart Fail. 2024;26(8):1830–1. 10.1002/ejhf.3331 38874148

[62] Gupta P, et al. Forecasting heart failure: Seasonal alignment of heart failure outcomes in New York. ESC Heart Fail. 2025;12(2):819–31. 10.1002/ehf2.14964 PMC11911598 39549264

[63] Vanasse A, et al. Effects of climate and fine particulate matter on hospitalizations and deaths for heart failure in elderly: A population-based cohort study. Environ Int. 2017;106:257–66. 10.1016/j.envint.2017.06.001 28709636

[64] Lamas GA, et al. Contaminant Metals as Cardiovascular Risk Factors: A Scientific Statement From the American Heart Association. J Am Heart Assoc. 2023;12(13):e029852. 10.1161/JAHA.123.029852 PMC10356104 37306302

[65] Cheema S, et al. Toxic heavy metal exposure and heart health: a systematic review and meta-analysis of 324,331 patients. BMC Cardiovasc Disord. 2025;25(1):792. 10.1186/s12872-025-05248-9 PMC12595707 41204397

[66] Borne Y, et al. Cadmium exposure and incidence of heart failure and atrial fibrillation: a population-based prospective cohort study. BMJ Open. 2015;5(6):e007366. 10.1136/bmjopen-2014-007366 PMC4480021 26078311

[67] Sears CG, et al. Urinary Cadmium and Incident Heart Failure: A Case-Cohort Analysis Among Never-Smokers in Denmark. Epidemiology. 2022;33(2):185–92. 10.1097/EDE.0000000000001446 PMC8810592 34860726

[68] Martinez-Morata I, et al. Associations Between Urinary Metal Levels and Incident Heart Failure: A Multi-Cohort Analysis. JACC Heart Fail. 2025;13(8):102510. 10.1016/j.jchf.2025.03.046 PMC12284413 40532446

[69] Peters JL, et al. Cadmium exposure in association with history of stroke and heart failure. Environ Res. 2010;110(2):199–206. 10.1016/j.envres.2009.12.004 PMC3031174 20060521

[70] Lin Z, et al. Association between heavy metal exposure and heart failure incidence and mortality: insights from NHANES data (2003–2018). BMC Public Health. 2025;25(1):1935. 10.1186/s12889-025-23177-2 PMC12105153 40420024

[71] Yuting Y, Shan D. Associations between urinary and blood heavy metal exposure and heart failure in elderly adults: Insights from an interpretable machine learning model based on NHANES (2003–2020). Int J Cardiol Cardiovasc Risk Prev. 2025;25:200418. 10.1016/j.ijcrp.2025.200418 PMC12146108 40491714

[72] Deering KE, et al. Low-level cadmium exposure and cardiovascular outcomes in elderly Australian women: A cohort study. Int J Hyg Environ Health. 2018;221(2):347–54. 10.1016/j.ijheh.2017.12.007 29331275

[73] Pichler G, et al. Association of Arsenic Exposure With Cardiac Geometry and Left Ventricular Function in Young Adults. Circ Cardiovasc Imaging. 2019;12(5):e009018. 10.1161/CIRCIMAGING.119.009018 PMC6668025 31060373

[74] Lieberman-Cribbin W, et al. Relationship Between Urinary Uranium and Cardiac Geometry and Left Ventricular Function: The Strong Heart Study. JACC Adv. 2024;3(12):101408. 10.1016/j.jacadv.2024.101408 PMC11617505 39640231

[75] Yang WY et al. Left Ventricular Structure and Function in Relation to Environmental Exposure to Lead and Cadmium. J Am Heart Assoc, 2017. 6(2). 10.1161/JAHA.116.004692 PMC5523767 28151401

[76] Zago AM, et al. Pesticide exposure and risk of cardiovascular disease: A systematic review. Glob Public Health. 2022;17(12):3944–66. 10.1080/17441692.2020.1808693 32816635

[77] Mohammadkhani MA, et al. Insights into Organochlorine Pesticides Exposure in the Development of Cardiovascular Diseases: A Systematic Review. Arch Iran Med. 2023;26(10):592–9. 10.34172/aim.2023.86 PMC10862093 38310416

[78] Akesson A, et al. Dietary exposure to polychlorinated biphenyls and risk of heart failure - A population-based prospective cohort study. Environ Int. 2019;126:1–6. 10.1016/j.envint.2019.01.069 30776745

[79] Hung DZ, et al. The Long-Term Effects of Organophosphates Poisoning as a Risk Factor of CVDs: A Nationwide Population-Based Cohort Study. PLoS ONE. 2015;10(9):e0137632. 10.1371/journal.pone.0137632 PMC4560399 26339906

[80] Sjoberg Lind Y, et al. Persistent organic pollutants and abnormal geometry of the left ventricle in the elderly. J Hypertens. 2013;31(8):1547–53. 10.1097/HJH.0b013e32836221b3 23743808

[81] Lind PM, et al. Circulating levels of p,p’-DDE are related to prevalent hypertension in the elderly. Environ Res. 2014;129:27–31. 10.1016/j.envres.2013.12.003 24528999

[82] Donat-Vargas C, et al. Association between dietary intake of polychlorinated biphenyls and the incidence of hypertension in a Spanish cohort: the Seguimiento Universidad de Navarra project. Hypertension. 2015;65(4):714–21. 10.1161/HYPERTENSIONAHA.114.04435 25646299

[83] Calderon L, et al. Residential proximity to agricultural pesticide use and cardiovascular disease risk factors among adult Latina women in California’s Salinas Valley. Am J Epidemiol. 2024;193(11):1583–91. 10.1093/aje/kwae118 PMC12107027 38872348

[84] Mills KT, et al. Pesticides and myocardial infarction incidence and mortality among male pesticide applicators in the Agricultural Health Study. Am J Epidemiol. 2009;170(7):892–900. 10.1093/aje/kwp214 PMC2765359 19700503

[85] Dayton SB, et al. Pesticide use and myocardial infarction incidence among farm women in the agricultural health study. J Occup Environ Med. 2010;52(7):693–7. 10.1097/JOM.0b013e3181e66d25 PMC2927225 20595914

[86] Zhao X, et al. A prospective cohort study of exposure to household pesticide with cardiovascular diseases mortality in older adults. J Hazard Mater. 2024;471:134316. 10.1016/j.jhazmat.2024.134316 38669923

[87] Xue Q, et al. Association between pyrethroid exposure and cardiovascular disease: A national population-based cross-sectional study in the US. Environ Int. 2021;153:106545. 10.1016/j.envint.2021.106545 33839550

[88] Gamble KL, et al. Circadian clock control of endocrine factors. Nat Rev Endocrinol. 2014;10(8):466–75. 10.1038/nrendo.2014.78 PMC4304769 24863387

[89] Cribbet MR, et al. Circadian rhythms and metabolism: from the brain to the gut and back again. Ann N Y Acad Sci. 2016;1385(1):21–40. 10.1111/nyas.13188 PMC5428740 27589593

[90] Masri S, Sassone-Corsi P. The circadian clock: a framework linking metabolism, epigenetics and neuronal function. Nat Rev Neurosci. 2013;14(1):69–75. 10.1038/nrn3393 PMC5720680 23187814

[91] Li X, et al. Chronobiological disruptions: unravelling the interplay of shift work, circadian rhythms, and vascular health in the context of stroke risk. Clin Exp Med. 2024;25(1):6. 10.1007/s10238-024-01514-w PMC11564290 39541048

[92] Figueiro M, Overington D. Self-luminous devices and melatonin suppression in adolescents. Lighting Res Technol. 2016;48(8):966–75.

[93] Lunn RM, et al. Health consequences of electric lighting practices in the modern world: A report on the National Toxicology Program’s workshop on shift work at night, artificial light at night, and circadian disruption. Sci Total Environ. 2017;607–608:1073–84. 10.1016/j.scitotenv.2017.07.056 PMC5587396 28724246

[94] Huang T. Sleep Irregularity, Circadian Disruption, and Cardiometabolic Disease Risk. Circ Res. 2025;137(5):709–26. 10.1161/CIRCRESAHA.125.325613 PMC12356488 40811504

[95] Cain SW, et al. Evening home lighting adversely impacts the circadian system and sleep. Sci Rep. 2020;10(1):19110. 10.1038/s41598-020-75622-4 PMC7644684 33154450

[96] Lane KJ, et al. Associations between Greenness, Impervious Surface Area, and Nighttime Lights on Biomarkers of Vascular Aging in Chennai, India. Environ Health Perspect. 2017;125(8):087003. 10.1289/EHP541 PMC5783666 28886599

[97] Zhang J, et al. Role of Nighttime Light in the Association Between Air Pollution Exposure and Cardiovascular Disease. J Am Heart Assoc. 2025;14(16):e042835. 10.1161/JAHA.125.042835 PMC12748063 40767279

[98] Windred DP, et al. Light Exposure at Night and Cardiovascular Disease Incidence. JAMA Netw Open. 2025;8(10):e2539031. 10.1001/jamanetworkopen.2025.39031 PMC12550636 41129148

[99] Schultz WM, et al. Socioeconomic Status and Cardiovascular Outcomes: Challenges and Interventions. Circulation. 2018;137(20):2166–78. 10.1161/CIRCULATIONAHA.117.029652 PMC5958918 29760227

[100] Xia N, Li H. Loneliness, Social Isolation, and Cardiovascular Health. Antioxid Redox Signal. 2018;28(9):837–51. 10.1089/ars.2017.7312 PMC5831910 28903579

[101] Liang YY, et al. Association of Social Isolation and Loneliness With Incident Heart Failure in a Population-Based Cohort Study. JACC Heart Fail. 2023;11(3):334–44. 10.1016/j.jchf.2022.11.028 PMC9891238 36737310

[102] Valtorta NK, et al. Loneliness and social isolation as risk factors for coronary heart disease and stroke: systematic review and meta-analysis of longitudinal observational studies. Heart. 2016;102(13):1009–16. 10.1136/heartjnl-2015-308790 PMC4941172 27091846

[103] Hashem IEM, Hamid MNMA, Abdou HA, S Al-Kindi, S Abohashem, Pasha LI, et al. Loneliness and cardiovascular disease: a narrative review of epidemiology, mechanisms, and clinical implications. Heart Mind. 2026;10(2):126–39. 10.4103/hm.HM-D-26-00020.

[104] Shakoor A, et al. Socio-economic inequalities and heart failure morbidity and mortality: A systematic review and data synthesis. ESC Heart Fail. 2025;12(2):927–41. 10.1002/ehf2.14986 PMC11911605 39318286

[105] Kwaah PA, et al. Impact of County-Level Social Disadvantage Indices on Heart Failure Hospitalizations in the United States. J Cardiac Fail - Intersections. 2026;2(3):274–7.

[106] Benito T, et al. Risk factors and prognostic impact of unwanted loneliness in heart failure. Sci Rep. 2024;14(1):22229. 10.1038/s41598-024-72847-5 PMC11436651 39333672

[107] Paul E, Bu F, Fancourt D. Loneliness and Risk for Cardiovascular Disease: Mechanisms and Future Directions. Curr Cardiol Rep. 2021;23(6):68. 10.1007/s11886-021-01495-2 PMC8105233 33961131

[108] Celano CM, et al. Depression and Anxiety in Heart Failure: A Review. Harv Rev Psychiatry. 2018;26(4):175–84. 10.1097/HRP.0000000000000162 PMC6042975 29975336

[109] Wu JR, Moser DK. Medication Adherence Mediates the Relationship Between Heart Failure Symptoms and Cardiac Event-Free Survival in Patients With Heart Failure. J Cardiovasc Nurs. 2018;33(1):40–6. 10.1097/JCN.0000000000000427 PMC5714687 28591004

[110] Chen H, et al. Residential Greenness and Cardiovascular Disease Incidence, Readmission, and Mortality. Environ Health Perspect. 2020;128(8):87005. 10.1289/EHP6161 PMC7446772 32840393

[111] Makhlouf MHE, et al. Neighborhood Walkability and Cardiovascular Risk in the United States. Curr Probl Cardiol. 2023;48(3):101533. 10.1016/j.cpcardiol.2022.101533 PMC9892210 36481391

[112] Meijer P, et al. Changes in neighbourhood walkability and incident CVD: A population-based cohort study of three million adults covering 24 years. Environ Res. 2025;274:121367. 10.1016/j.envres.2025.121367 40073923

[113] Bober T, et al. Impact of Neighborhood Factors on Heart Failure Outcomes: Secondary Analysis From the Hopeful Heart Trial. JACC Adv. 2025;4(10 Pt 2):102146. 10.1016/j.jacadv.2025.102146 PMC12464708 40953541

[114] Ajufo E, et al. Accelerometer-Measured Sedentary Behavior and Risk of Future Cardiovascular Disease. J Am Coll Cardiol. 2025;85(5):473–86. 10.1016/j.jacc.2024.10.065 PMC11851520 39545903

[115] Echouffo-Tcheugui JB, et al. Association of Physical Activity or Fitness With Incident Heart Failure: A Systematic Review and Meta-Analysis. Circ Heart Fail. 2015;8(5):853–61. 10.1161/CIRCHEARTFAILURE.115.002070 26175539

[116] Ho FK, et al. Association Between Device-Measured Physical Activity and Incident Heart Failure: A Prospective Cohort Study of 94 739 UK Biobank Participants. Circulation. 2022;146(12):883–91. 10.1161/CIRCULATIONAHA.122.059663 36036153

[117] Piepoli MF, et al. Exercise training meta-analysis of trials in patients with chronic heart failure (ExTraMATCH). BMJ. 2004;328(7433):189. 10.1136/bmj.37938.645220.EE PMC318480 14729656

[118] Hua C, et al. Effects of exercise based cardiac rehabilitation delivery modes on chronic heart failure: a systematic review and network meta-analysis. Sci Rep. 2024;14(1):31246. 10.1038/s41598-024-82608-z PMC11682296 39732888

[119] Florido R, et al. Physical Activity and Incident Heart Failure in High-Risk Subgroups: The ARIC Study. J Am Heart Assoc. 2020;9(10):e014885. 10.1161/JAHA.119.014885 PMC7660876 32390492

[120] Cacciatore F, et al. Protective effect of physical activity on mortality in older adults with advanced chronic heart failure: A prospective observational study. Eur J Prev Cardiol. 2019;26(5):481–8. 10.1177/2047487318790822 30066588

[121] Coats AJS, et al. Physical function and exercise training in older patients with heart failure. Nat Rev Cardiol. 2017;14(9):550–9. 10.1038/nrcardio.2017.70 PMC7245611 28518178

[122] Lara KM, et al. Dietary Patterns and Incident Heart Failure in U.S. Adults Without Known Coronary Disease. J Am Coll Cardiol. 2019;73(16):2036–45. 10.1016/j.jacc.2019.01.067 PMC6501554 31023426

[123] Levitan EB, Wolk A, Mittleman MA. Consistency with the DASH diet and incidence of heart failure. Arch Intern Med. 2009;169(9):851–7. 10.1001/archinternmed.2009.56 PMC2682222 19433696

[124] Chiavaroli L et al. DASH Dietary Pattern and Cardiometabolic Outcomes: An Umbrella Review of Systematic Reviews and Meta-Analyses. Nutrients. 2019;11(2). 10.3390/nu11020338 PMC6413235 30764511

[125] Butler T. Dietary management of heart failure: room for improvement? Br J Nutr. 2016;115(7):1202–17. 10.1017/S000711451500553X 26857032

[126] Wickman BE et al. Dietary Management of Heart Failure: DASH Diet and Precision Nutrition Perspectives. Nutrients. 2021;13(12). 10.3390/nu13124424 PMC8708696 34959976

[127] Wleklik M, et al. The Role of Nutritional Status in Elderly Patients with Heart Failure. J Nutr Health Aging. 2018;22(5):581–8. 10.1007/s12603-017-0985-1 PMC12876321 29717757

[128] Rahman A, et al. Malnutrition and Cachexia in Heart Failure. JPEN J Parenter Enter Nutr. 2016;40(4):475–86. 10.1177/0148607114566854 25634161

[129] Catapano G, et al. Nutrient intake and serum cytokine pattern in elderly people with heart failure. Eur J Heart Fail. 2008;10(4):428–34. 10.1016/j.ejheart.2008.02.016 18353717

[130] Vasan RS, et al. Lifetime Risk of Heart Failure Among Participants in the Framingham Study. J Am Coll Cardiol. 2022;79(3):250–63. 10.1016/j.jacc.2021.10.043 PMC8820638 35057911

[131] Goyal P, Maurer MS, Roh J. Aging in Heart Failure: Embracing Biology Over Chronology: JACC Family Series. JACC Heart Fail. 2024;12(5):795–809. 10.1016/j.jchf.2024.02.021 PMC11331491 38597865

[132] Vakka A, Warren JS, Drosatos K. Cardiovascular aging: from cellular and molecular changes to therapeutic interventions. J Cardiovasc Aging 2023;3(3). 10.20517/jca.2023.09 PMC10238104 37274126

[133] Tana M, et al. Cardiovascular Aging Rev Cardiovasc Med. 2025;26(7):27437. 10.31083/RCM27437 PMC12326460 40776937

[134] McMurray J, et al. Evidence of oxidative stress in chronic heart failure in humans. Eur Heart J. 1993;14(11):1493–8. 10.1093/eurheartj/14.11.1493 8299631

[135] Ungvari Z, et al. Age-associated vascular oxidative stress, Nrf2 dysfunction, and NF-kappaB activation in the nonhuman primate Macaca mulatta. J Gerontol Biol Sci Med Sci. 2011;66(8):866–75. 10.1093/gerona/glr092 PMC3148762 21622983

[136] Daiber A, et al. Cardiovascular risk posed by the exposome. Atherosclerosis. 2025;405:119222. 10.1016/j.atherosclerosis.2025.119222 40339362

[137] Mlynarska E et al. Inflammaging and Senescence-Driven Extracellular Matrix Remodeling in Age-Associated Cardiovascular Disease. Biomolecules. 2025;15(10). 10.3390/biom15101452 PMC12564436 41154680

[138] Spray L, et al. Cardiovascular inflammaging: Mechanisms, consequences, and therapeutic perspectives. Cell Rep Med. 2025;6(9):102264. 10.1016/j.xcrm.2025.102264 PMC12490256 40782796

[139] Barcena ML et al. Cardiovascular Inflammaging: Mechanisms and Translational Aspects. Cells. 2022;11(6). 10.3390/cells11061010 PMC8946971 35326461

[140] Qiu H, Gladysheva IP. Therapeutic perspective on cardiomyopathy and heart failure in older adults through the lens of chronic inflammation. Vascul Pharmacol. 2026;162:107586. 10.1016/j.vph.2026.107586 PMC13201211 41702467

[141] Abdellatif M, et al. Hallmarks of cardiovascular ageing. Nat Rev Cardiol. 2023;20(11):754–77. 10.1038/s41569-023-00881-3 37193857

[142] Santovito D, et al. Autophagy, innate immunity, and cardiac disease. Front Cell Dev Biol. 2023;11:1149409. 10.3389/fcell.2023.1149409 PMC10206260 37234771

[143] Maldonado E et al. Aging Hallmarks and the Role of Oxidative Stress. Antioxid (Basel). 2023;12(3). 10.3390/antiox12030651 PMC10044767 36978899

[144] Chang ACY, et al. Telomere shortening is a hallmark of genetic cardiomyopathies. Proc Natl Acad Sci U S A. 2018;115(37):9276–81. 10.1073/pnas.1714538115 PMC6140486 30150400

[145] Bhayadia R, et al. Senescence-Induced Oxidative Stress Causes Endothelial Dysfunction. J Gerontol Biol Sci Med Sci. 2016;71(2):161–9. 10.1093/gerona/glv008 25735595

[146] Coppe JP, et al. The senescence-associated secretory phenotype: the dark side of tumor suppression. Annu Rev Pathol. 2010;5:99–118. 10.1146/annurev-pathol-121808-102144 PMC4166495 20078217

[147] Rouhi L, et al. Haploinsufficiency of Tmem43 in cardiac myocytes activates the DNA damage response pathway leading to a late-onset senescence-associated pro-fibrotic cardiomyopathy. Cardiovasc Res. 2021;117(11):2377–94. 10.1093/cvr/cvaa300 PMC8861264 33070193

[148] Lin YK, et al. Aging Modulates the Substrate and Triggers Remodeling in Atrial Fibrillation. Circ J. 2018;82(5):1237–44. 10.1253/circj.CJ-17-0242 28904308

[149] Xu C, et al. The role of cellular senescence in cardiovascular disease. Cell Death Discov. 2025;11(1):431. 10.1038/s41420-025-02720-5 PMC12501035 41053058

[150] Zhu FL et al. Senescent Cardiac Fibroblast Is Critical for Cardiac Fibrosis after Myocardial Infarction. PLoS ONE. 2013;8(9). 10.1371/journal.pone.0074535 PMC3770549 24040275

[151] Redgrave RE et al. Senescent cardiomyocytes contribute to cardiac dysfunction following myocardial infarction. Npj Aging. 2023;9(1):15. 10.1038/s41514-023-00113-5 PMC10267185 37316516

[152] Gevaert AB et al. Endothelial Senescence Contributes to Heart Failure With Preserved Ejection Fraction in an Aging Mouse Model. Circ Heart Fail. 2017;10(6). 10.1161/CIRCHEARTFAILURE.116.003806 28611124

[153] Sharma K, et al. Fine air pollution particles induce endothelial senescence via redox-sensitive activation of local angiotensin system. Environ Pollut. 2019;252(Pt A):317–29. 10.1016/j.envpol.2019.05.066 31158660

[154] Munzel T, et al. Transportation Noise Pollution and Cardiovascular Health. Circ Res. 2024;134(9):1113–35. 10.1161/CIRCRESAHA.123.323584 38662856

[155] Munzel T, et al. The Adverse Effects of Environmental Noise Exposure on Oxidative Stress and Cardiovascular Risk. Antioxid Redox Signal. 2018;28(9):873–908. 10.1089/ars.2017.7118 PMC5898791 29350061

[156] Zuurbier LA, et al. Fragmentation and stability of circadian activity rhythms predict mortality: the Rotterdam study. Am J Epidemiol. 2015;181(1):54–63. 10.1093/aje/kwu245 25491893

[157] Nathan P, et al. Changes in Circadian Rhythms Dysregulate Inflammation in Ageing: Focus on Leukocyte Trafficking. Front Immunol. 2021;12:673405. 10.3389/fimmu.2021.673405 PMC8160305 34054857

[158] Schulte W, Heusch G, von Eiff AW. The influence of experimental traffic noise on autonomous functions of normotensives and hypertensives after stress (author’s transl)]. Basic Res Cardiol. 1977;72(6):575–83. 10.1007/BF01907038 607927

[159] Goligorsky MS. Permissive role of vascular endothelium in fibrosis: focus on the kidney. Am J Physiol Cell Physiol. 2024;326(3):C712–23. 10.1152/ajpcell.00526.2023 PMC11193458 38223932

[160] Radakrishnan A, et al. Underpinnings of heart failure with preserved ejection fraction in women - From prevention to improving function. A co-publication with the American Journal of Preventive Cardiology and the Journal of Cardiac Failure. Am J Prev Cardiol. 2025;23:100928. 10.1016/j.ajpc.2025.100928 PMC12354818 40823618

[161] de Almeida A, et al. Unveiling the Role of Inflammation and Oxidative Stress on Age-Related Cardiovascular Diseases. Oxid Med Cell Longev. 2020;2020:1954398. 10.1155/2020/1954398 PMC7232723 32454933

[162] Kuntic M, et al. Impact of air pollution on cardiovascular aging. Mech Ageing Dev. 2023;214:111857. 10.1016/j.mad.2023.111857 37611809

[163] Fonseka O et al. Molecular Mechanisms Underlying Heart Failure and Their Therapeutic Potential. Cells. 2025;14(5). 10.3390/cells14050324 PMC11899429 40072053

[164] Kishi S, et al. Zebrafish as a genetic model in biological and behavioral gerontology: where development meets aging in vertebrates–a mini-review. Gerontology. 2009;55(4):430–41. 10.1159/000228892 PMC2820570 19654474

[165] El Mai M et al. Opposing p53 and mTOR/AKT promote an in vivo switch from apoptosis to senescence upon telomere shortening in zebrafish. Elife. 2020;9. 10.7554/eLife.54935 PMC7237213 32427102

[166] Martins RR, et al. Telomerase Depletion Accelerates Ageing of the Zebrafish Brain. Aging Cell. 2025;24(12):e70280. 10.1111/acel.70280 PMC12686574 41147684

[167] Bevan L, et al. Aged Zebrafish as a Spontaneous Model of Cardiac Valvular Disease. Aging Cell. 2025;24(12):e70266. 10.1111/acel.70266 PMC12686566 41077830

[168] Poss KD, Wilson LG, Keating MT. Heart regeneration in zebrafish. Science. 2002;298(5601):2188–90. 10.1126/science.1077857 12481136

[169] Reuter H et al. Aging Activates the Immune System and Alters the Regenerative Capacity in the Zebrafish Heart. Cells. 2022;11(3). 10.3390/cells11030345 PMC8834511 35159152

[170] Coppola A et al. Zebrafish as a Model of Cardiac Pathology and Toxicity: Spotlight on Uremic Toxins. Int J Mol Sci. 2023;24(6). 10.3390/ijms24065656 PMC10052014 36982730

[171] Birse RT, et al. High-fat-diet-induced obesity and heart dysfunction are regulated by the TOR pathway in Drosophila. Cell Metab. 2010;12(5):533–44. 10.1016/j.cmet.2010.09.014 PMC3026640 21035763

[172] Piper MDW, Partridge L. Drosophila as a model for ageing. Biochim Biophys Acta Mol Basis Dis. 2018;1864(9 Pt A):2707–17. 10.1016/j.bbadis.2017.09.016 28964875

[173] Ogienko AA et al. Drosophila as a Model Organism to Study Basic Mechanisms of Longevity. Int J Mol Sci. 2022;23(19). 10.3390/ijms231911244 PMC9569508 36232546

[174] Lian T, et al. Epigenetic mechanisms of dietary restriction induced aging in Drosophila. Exp Gerontol. 2015;72:38–44. 10.1016/j.exger.2015.08.015 26342637

[175] Shukla N, Kolthur-Seetharam U. Drosophila Sirtuin 6 mediates developmental diet-dependent programming of adult physiology and survival. Aging Cell. 2022;21(3):e13576. 10.1111/acel.13576 PMC8920434 35233942

[176] Neely GG, et al. A global in vivo Drosophila RNAi screen identifies NOT3 as a conserved regulator of heart function. Cell. 2010;141(1):142–53. 10.1016/j.cell.2010.02.023 PMC2855221 20371351

[177] Ping X, et al. Hypoxic compound exercise improves cardiac function in Drosophila high fructose diet via KHK. J Mol Cell Cardiol. 2025;201:95–104. 10.1016/j.yjmcc.2025.02.005 39954938

[178] Ocorr K, et al. Genetic control of heart function and aging in Drosophila. Trends Cardiovasc Med. 2007;17(5):177–82. 10.1016/j.tcm.2007.04.001 PMC1950717 17574126

[179] Lovato TL et al. Modeling a variant of unknown significance in the Drosophila ortholog of the human cardiogenic gene NKX2.5. Dis Model Mech. 2023;16(9). 10.1242/dmm.050059 PMC10548113 37691628

[180] Souidi A, Jagla K. Drosophila Heart as a Model for Cardiac Development and Diseases. Cells. 2021;10(11). 10.3390/cells10113078 PMC8623483 34831301

